# Research Advances and Perspectives on Gut Microbiota Regulating Alzheimer’s Disease

**DOI:** 10.3390/biology15201893

**Published:** 2026-10-20

**Authors:** Fanfan Zhu, Xiaoyi Shao

**Affiliations:** 1 Clinical Medicine, Medical School, Nantong University, Nantong 226001, China; zhufanfan@stmail.ntu.edu.cn or 19825283625@163.com; 2 Department of Immunology, Medical School, Xinglin College, Nantong University, Nantong 226001, China

**Keywords:** Alzheimer’s disease, gut microbiota, gut-microbiota-brain axis, amyloid β-protein, short-chain fatty acids, microbiota dysbiosis

## Abstract

Alzheimer’s disease is a neurodegenerative disease characterized by progressive cognitive impairment. One of the primary reasons for the significant challenges in its clinical management is that the pathogenesis of Alzheimer’s disease has not been fully elucidated and effective intervention methods remain lacking. Therefore, it is crucial to explore relevant peripheral risk factors and novel therapeutic targets. The gut–microbiota–brain axis is a complex bidirectional regulatory network that integrates neural, endocrine, immune, and metabolic pathways. As the core of this axis, the gut microbiota modulates neuroinflammation, synaptic plasticity, and protein homeostasis. Specifically, metabolites such as short-chain fatty acids can cross the blood–brain barrier to regulate microglia activation, while microbial-mediated immunomodulatory effects directly influence the deposition of amyloid-β and the phosphorylation of tau protein. Existing studies have confirmed that Alzheimer’s disease patients exhibit gut microbiota imbalance, typically manifested as a decreased abundance of beneficial bacteria and the proliferation of potential proinflammatory bacteria. The dysbiosis is closely related to the accumulation of core pathological markers and the severity of cognitive impairment. This article aims to elucidate the interaction and underlying mechanisms between the gut microbiota and Alzheimer’s disease, offering an integrated analysis and research prospects to facilitate the development of novel intervention strategies.

## Simple Summary

This review focuses on gut microbiota as a peripheral factor in Alzheimer’s disease, a condition with limited treatment options and a growing global burden. While accumulating evidence suggests that gut microbes can send signals to the brain and influence disease progression, most current studies only show correlations. We summarize this evidence and discuss the practical challenges of translating laboratory findings into clinical applications. Further well-designed human studies are needed to determine whether gut microbiota can be used for early screening or therapeutic intervention in Alzheimer’s disease.

## 1. Introduction

Alzheimer’s disease (AD) is a neurodegenerative disease characterized by progressive cognitive impairment. Its core pathological features include amyloid-β(Aβ) deposition, abnormal aggregation of Tau protein, and chronic neuroinflammation, which imposes a heavy burden on societies and families worldwide [1]. Currently, clinical treatments are limited and conventional drugs can only alleviate symptoms, while therapies targeting core pathological hallmarks show inconsistent effects and pose safety risks. Consequently, exploring novel intervention targets is urgently needed [2,3].

The gut–microbiota–brain axis represents a bidirectional regulatory network connecting the periphery and the central nervous system (CNS), making the gut microbiota a new focus in AD research [4]. Numerous studies have confirmed that patients with AD exhibit significant gut microbiota dysbiosis, characterized by decreased abundance of beneficial bacteria (e.g., *Faecalibacterium*) and proliferation of proinflammatory bacteria. This dysbiosis is closely associated with the deposition of AD pathological markers and cognitive impairment, and can even be detected in the preclinical stage of the disease [5,6,7,8].

**Figure 1:**
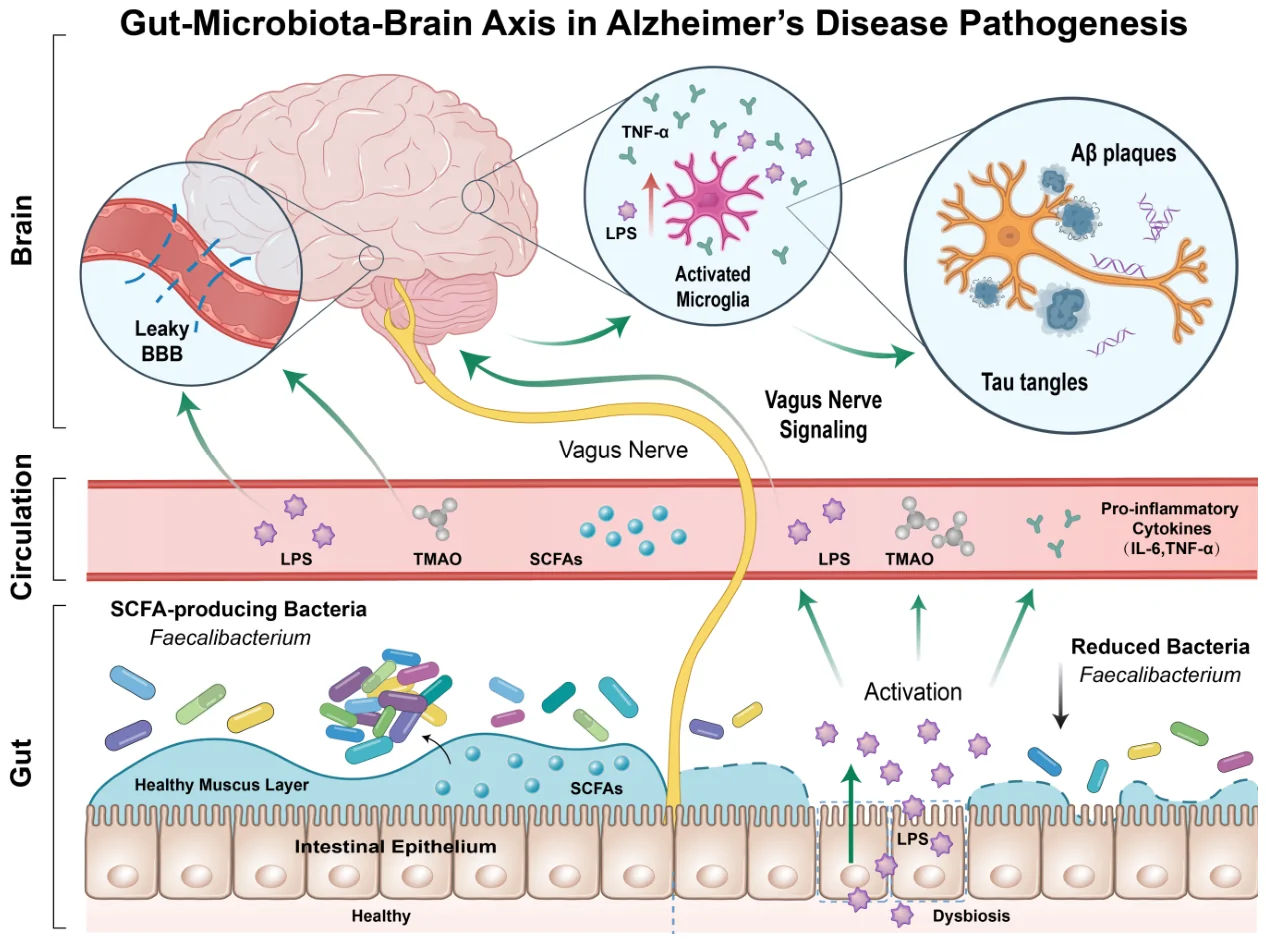
The gut–microbiota–brain axis contributes to the pathogenesis of Alzheimer’s disease. Schematic diagram illustrating that gut dysbiosis induces systemic inflammation and metabolic disorder, disrupts the blood–brain barrier, triggers central neuroinflammation, and ultimately promotes Aβ deposition, tau hyperphosphorylation, and neuronal damage. Abbreviations: Aβ, amyloid-β; BBB, blood–brain barrier; IL-6, interleukin-6; LPS, lipopolysaccharide; SCFAs, short-chain fatty acids; TMAO, trimethylamine N-oxide; TNF-α, tumor necrosis factor-α. Created by the author.

This review aims to summarize the association between gut microbiota and AD as well as recent research advances. It also depicts the core pathways by which gut microbiota modulates AD, evaluates the intervention potential of targeting gut microbiota, and discusses the challenges and future directions [9,10,11,12,13] (Figure 1).

## 2. Search Strategy

This is a narrative review, and no formal systematic review protocol was implemented. Literature searches were performed in PubMed and Web of Science for articles published between 2016 and December 2025. Search terms included combinations of the following keywords: “gut microbiota”, “gut–microbiota–brain axis”, “Alzheimer’s disease”, “amyloid-β”, “tau”, “cognitive impairment”, “neuroinflammation”, “short-chain fatty acids”, “lipopolysaccharide”, “dysbiosis”, “intestinal permeability”, “microglia”, and “neurodegeneration”. Publications were selected based on the relevance of their titles and abstracts to the aim of this review and their coverage of gut microbiota, gut metabolites, gut-microbiota-brain axis signaling, and AD-related pathological or cognitive phenotypes. Publications outside the scope of this review, editorials, case reports, and conference abstracts were excluded. All retrieved records were screened manually.

## 3. Current Research Status of AD

The global morbidity of AD continues to rise with population aging and the disease shows regional and ethnic variations. Accordingly, AD’s burden on society and economy increases progressively [1,14,15]. Core pathological hallmarks of AD include Aβ deposition, abnormal aggregation of Tau protein, and chronic neuroinflammation. Vascular dysfunction also constitutes a key early event of the disease. The Aβ cascade hypothesis proposes that extracellular senile plaques formed by an imbalance between production and clearance of Aβ_42_ peptides are critical to disease progression, while neurofibrillary tangles composed of hyperphosphorylated Tau protein disrupt neuronal function. Meanwhile, Aβ pathology typically precedes and exacerbates Tau pathology. Both processes can interact with neuroinflammation, and chronic neuroinflammation may further damage neurons [16,17,18]. Loss of blood–brain barrier integrity accelerates the accumulation of toxic substances, and disrupted microbial homeostasis directly reduces the clearance efficiency of metabolic wastes derived from Aβ in the central lymphatic system. These mechanisms interact with synaptic dysfunction, neuronal death, and systemic metabolic disorders to collectively promote disease progression [1,19,20].

Current treatments for AD mainly comprise symptomatic therapy, disease-modifying therapies, and lifestyle interventions [21]. Symptomatic drugs such as traditional cholinesterase inhibitors only moderately alleviate symptoms, fail to block disease progression, and cause side effects [2,22]. In recent years, disease-modifying therapies have shifted toward targeting core pathological features, yet clinical trial results of Aβ-targeting monoclonal antibodies vary [23]. For instance, lecanemab can eliminate cerebral Aβ plaques and delay cognitive decline in early-stage AD patients, and donanemab can slow disease progression [24,25]. In contrast, neither gantenerumab nor solanezumab achieved clinical benefits. Although the former reduced Aβ plaque load, it did not slow clinical deterioration, and the latter could not delay cognitive decline even with long-term therapy in preclinical AD populations [26,27]. Some drugs also carry safety risks of amyloid-related imaging abnormalities (ARIA). Intervention timing and target selection remain major challenges in AD treatment [28].

Currently, research is focused on emerging targets such as Tau protein targeting and neuroinflammation modulation, as well as risk factor management and healthy lifestyle interventions [6,17,23]. Specifically, adhering to a healthy diet and regular exercise can theoretically prevent or delay approximately 40% of dementia cases worldwide. Even in elderly individuals with existing AD pathological changes in the brain, maintaining a healthy lifestyle may better preserve cognitive function [15,29]. The cognitive-protective effects of a healthy diet can be mediated via the regulation of the gut microbiota [30,31]. Furthermore, the gut microbiota participates in AD pathological progression through the gut–microbiota–brain axis, and dysregulation of its composition and structure is closely related to neuroinflammation and Aβ deposition in the brain [7,13,32]. Therefore, gut–microbiota–brain axis targeted interventions have become a common research hotspot in the field of neurodegenerative diseases, yet their clinical translation requires further exploration [23,33,34].

## 4. Mechanisms Underlying the Involvement of Gut Microbiota in AD Pathogenesis

Recently, extensive evidence has demonstrated that gut microbiota communicate bidirectionally with the CNS through the gut–microbiota–brain axis, and their dysbiosis is closely linked to the onset and progression of AD.

### 4.1. Relevant Evidence Between AD and Gut Microbiota

Cross-sectional studies have consistently indicated marked differences in the composition, structure, and function of the gut microbiota between patients with AD and cognitively normal individuals [5,35,36,37]. These differences are reflected not only in reduced overall microbial diversity but also in decreased beneficial bacteria, including *Faecalibacterium* and *Roseburia*, along with increased abundance of potentially proinflammatory bacteria, such as *Escherichia* and *Shigella*. These beneficial bacteria possess anti-inflammatory properties and the ability to produce neuroprotective metabolites such as short-chain fatty acids (SCFAs) [38,39]. This pattern has been observed in studies across diverse populations, including Chinese cohorts of AD patients [35,40].

Notably, such compositional dysbiosis is statistically correlated with the core pathological features of AD. Early research found that the abundance of specific proinflammatory bacteria in the gut of patients was positively correlated with cerebral Aβ plaque burden detected by positron emission tomography (PET) and was associated with peripheral inflammation [7]. Research has indicated that in cognitively normal preclinical individuals with abnormal cerebrospinal fluid (CSF) Aβ/p-Tau biomarkers, distinct gut microbiota composition is significantly associated with AD pathological markers, suggesting an association between gut microbiota and the ATN (Aβ, Tau, Neurodegeneration) pathological framework [41]. A recent large-scale study using shallow shotgun metagenomics further identified that patients with AD show differences in gut microbial composition and functional pathways compared with cognitively normal individuals, and these differential signatures are directly associated with a comprehensive panel of ATN biomarkers spectrum in CSF, covering Aβ pathology, Tau pathology, neurodegeneration, and neuroinflammation [42]. Metagenomic studies have also reported that these AD-associated gut microbial signatures are disease-stage-specific and can distinguish individuals across different clinical stages of AD [5].

### 4.2. Gut Microbiota Signatures in Different Clinical Stages of AD

Gut microbiota alterations vary across the AD clinical spectrum, from preclinical AD through mild cognitive impairment (MCI), mild-to-moderate AD, and advanced AD dementia. These findings are inconsistent across published studies. Heterogeneity is most obvious in advanced AD dementia: some studies report progressive microbial changes, while others find no significant shifts (Table 1).

In the preclinical stage, when individuals exhibit Aβ pathology (Aβ-PET positive) but remain cognitively normal, gut microbiota dysbiosis can already be detected [43]. Studies have shown that the microbial composition of these asymptomatic individuals deviates significantly from healthy controls, with specific bacterial alterations related to inflammatory pathways like changes in the abundance of Bacteroides, suggesting that gut microbiota signatures may serve as early warning biomarkers for AD [43].

From the preclinical stage to the symptomatic phase, the association between microbiota dysbiosis and core pathology persists. A study covering the continuous spectrum from subjective cognitive decline to AD dementia found that the abundance of multiple SCFAs-producing beneficial bacteria like *Roseburia hominis* was reduced, which was significantly correlated with cerebrospinal fluid Aβ positivity and elevated phosphorylated Tau [41].

At the symptomatic stage, as disease progresses from mild cognitive impairment (MCI) to AD dementia, microbiota dysbiosis is further aggravated. It is characterized by a continuous decline in microbial diversity and progressive disorder of microbial functional pathways, such as the synthesis of SCFAs and tryptophan metabolism, which are critical for maintaining brain health [5,33,35]. At this time, the severity of microbiota dysbiosis shows a stronger correlation with patients’ declining cognitive test scores like Mini-Mental State Examination (MMSE) and Montreal Cognitive Assessment (MoCA), indicating parallel changes in gut–microbiota–brain axis perturbations and neurodegeneration, rather than establishing directional causality [35].

### 4.3. Key Factors Influencing the Association Between Gut Microbiota and AD

The association between gut microbiota and AD is modulated by a variety of host and environmental factors, which constitutes an important reason for the heterogeneity observed in current research findings.

Among host factors, the ApoEε4 genotype is the strongest known genetic risk factor [1,44]. Studies have revealed that ApoEε4 carriers display distinct gut microbiota characteristics, and this microbial composition significantly strengthens the association with cerebral Tau pathological burden [44]. Recent genetic colocalization studies provide mechanistic insights. Genome-wide association studies (GWAS) have demonstrated significant genetic correlations between AD risk loci and the abundance of specific gut microbiota, such as proinflammatory *Collinsella*. Enrichment of the proinflammatory *Collinsella* also shows a genetic correlation with the ApoEε4 risk allele [45]. This suggests that genetic background may modify individual susceptibility to AD and its pathological progression by shaping a specific proinflammatory gut microbiota ecosystem. Sex is another critical variable. Animal studies have confirmed that antibiotic-induced microbiome disturbance has selective, gender-specific effects on cerebral Aβ deposition and microglial homeostasis [46].

Environmental factors are equally crucial. Long-term Western-style diet, comorbid metabolic diseases such as type 2 diabetes, as well as extensive use of proton pump inhibitors, nonsteroidal anti-inflammatory drugs (NSAIDs) and other medications, can substantially alter the intestinal environment and modify the association between gut microbiota and AD [30,47,48,49].

In addition, heterogeneity in research methodologies cannot be ignored. Existing studies differ substantially in population selection (e.g., region, diagnostic criteria, disease stage) and methodology (e.g., sample processing, sequencing technology, and data analysis pipelines), which directly contribute to conflicts across study outcomes [36].

Specifically, these methodological differences can change the gut microbiota profiles detected in studies. Sequencing methods, including 16S rRNA gene sequencing and shotgun metagenomic sequencing, differ in their ability to identify bacterial species and microbial functions. Differences in sample handling, such as storage conditions, DNA extraction kits and primer choices, may bias measurements of microbial abundance. In addition, cohort features like age, medication, diet and concurrent illnesses also act as confounding factors. Across published studies, the finding that AD is linked to overall gut microbiota dysbiosis is more reliable than results about changes in single bacterial species [8,39].

### 4.4. Causality Between Gut Microbiota and AD

Most human studies employ a cross-sectional design. Although evidence for an association is substantial, it is difficult to distinguish whether gut microbiota dysbiosis plays a causal role in AD or merely represents a concomitant condition. Cross-sectional comparisons cannot establish progressive deterioration of the microbiota over the disease course, nor can they validate such microbial features as early diagnostic biomarkers. Strong causal evidence mainly comes from animal models. For instance, housing AD model mice in a germ-free environment results in cerebral Aβ pathology reducing substantially. But transplanting gut microbiota from AD mice into healthy germ-free mice can induce neuroinflammation and cognitive deficits in the recipients [13,50,51]. Cross-sectional gut-AD associations are further limited by confounders described in Section 4.3, hampering causal interpretation.

We separate three evidence types. Human observational studies show associations only. Animal studies support possible mechanisms. RCTs provide the strongest clinical evidence, though relevant AD microbiota trials are few.

Notably, reverse causation and residual confounding represent major interpretive challenges. AD-related changes, including shifts in dietary intake, altered medication use, slowed intestinal transit, and declines in daily functional capacity, may themselves reshape gut microbial composition and function, and these factors are often incompletely adjusted for in observational analyses.

As a complementary analytical method to tackle this challenge, Mendelian randomization provides a strategy to assess causal links between gut microbiota and AD. It helps reduce confounding effects common in conventional observational research. However, this method still carries limitations related to instrument variable selection and heterogeneity. Such methodological differences should be considered when interpreting relevant results.

Nevertheless, applying findings from animal models to humans should be done with caution. The key for future research depends on conducting large-scale, prospective population-based cohort studies alongside rigorously designed interventional trials. Only in this way can we clarify the potential causality between gut microbiota and AD and ultimately develop novel therapeutic strategies.

## 5. Core Mechanisms of How Gut Microbiota Regulates AD Pathogenesis

Gut microbiota influences the development of AD not through a single pathway, but via a multi-pathway, networking systemic mode. Gut microbiota and their metabolites can directly or indirectly regulate the homeostasis of the CNS through different routes, including metabolic, immune, neural, and endocrine pathways. These pathways mingle with each other and act collaboratively to jointly participate in the pathological progression of AD.

### 5.1. Metabolic Pathway: Bidirectional Regulatory Roles of Gut Microbiota Metabolites

Gut microbiota can convert dietary components into a variety of bioactive metabolites, which either reach distant organs by the circulatory system or act on enterochromaffin cells and nerve terminals in the intestinal lumen, thereby regulating host physiological and pathological processes [4]. In AD animal experiments, SCFAs, primarily consisting of butyrate, propionate, and acetate, are the major products of dietary fiber fermentation. They enter cells through monocarboxylic acid transport proteins such as monocarboxylate transporter 1 (MCT1) and solute carrier family 5 member 8 (SLC5A8) or act as ligands to activate G-protein-coupled receptors like GPR41/43 and GPR109A, exerting broad physiological functions [4,52]. These kinds of G-protein-coupled receptors are highly expressed in enteric neuroepithelial cells and are a core molecular target for SCFAs-mediated gut–microbiota–brain axis signaling [53].

In AD, the effects of SCFAs depend on different situations. On one hand, in animal models, as a histone deacetylase (HDAC, EC 3.5.1.98) inhibitor, butyrate can inhibit the nuclear factor-κB (NF-κB) pathway in microglia and downregulate the expression of proinflammatory factors such as IL-6 and tumor necrosis factor-α (TNF-α). Butyrate also enhances the expression of intestinal epithelial tight junction proteins like zonula occludens-1 (ZO-1) to maintain intestinal barrier integrity [54]. Animal studies strongly indicate that butyrate supplementation in AD models may directly target Aβ pathology. It can reduce Aβ production by downregulating β-site amyloid precursor protein cleaving enzyme 1 (BACE1, EC 3.4.23.46) expression, and meanwhile modulate microglial function to promote Aβ clearance, thereby synergistically alleviating plaque burden and improving cognitive function [54]. On the other hand, controversial studies suggest that in specific AD mouse models, such as APP/PS1, SCFAs, especially propionate, may promote the transformation of microglia into a pro-phagocytic phenotype, accelerating Aβ plaque deposition unexpectedly [33]. This indicates SCFAs’ effects may depend on multiple factors including local concentration, receptor expression levels, the disease stage, and host immune status.

These conflicting preclinical findings, which are primarily derived from animal models and cell-based mechanistic studies, can be attributed to variations in experimental design. Mouse genetic background and AD model strain, SCFA dosage, intervention duration, and disease stage at treatment onset collectively modulate microglial responses and amyloid pathology. Thus, consistent protective or detrimental outcomes of SCFAs cannot be generalized across all animal experimental settings [33,54].

**Figure 2:**
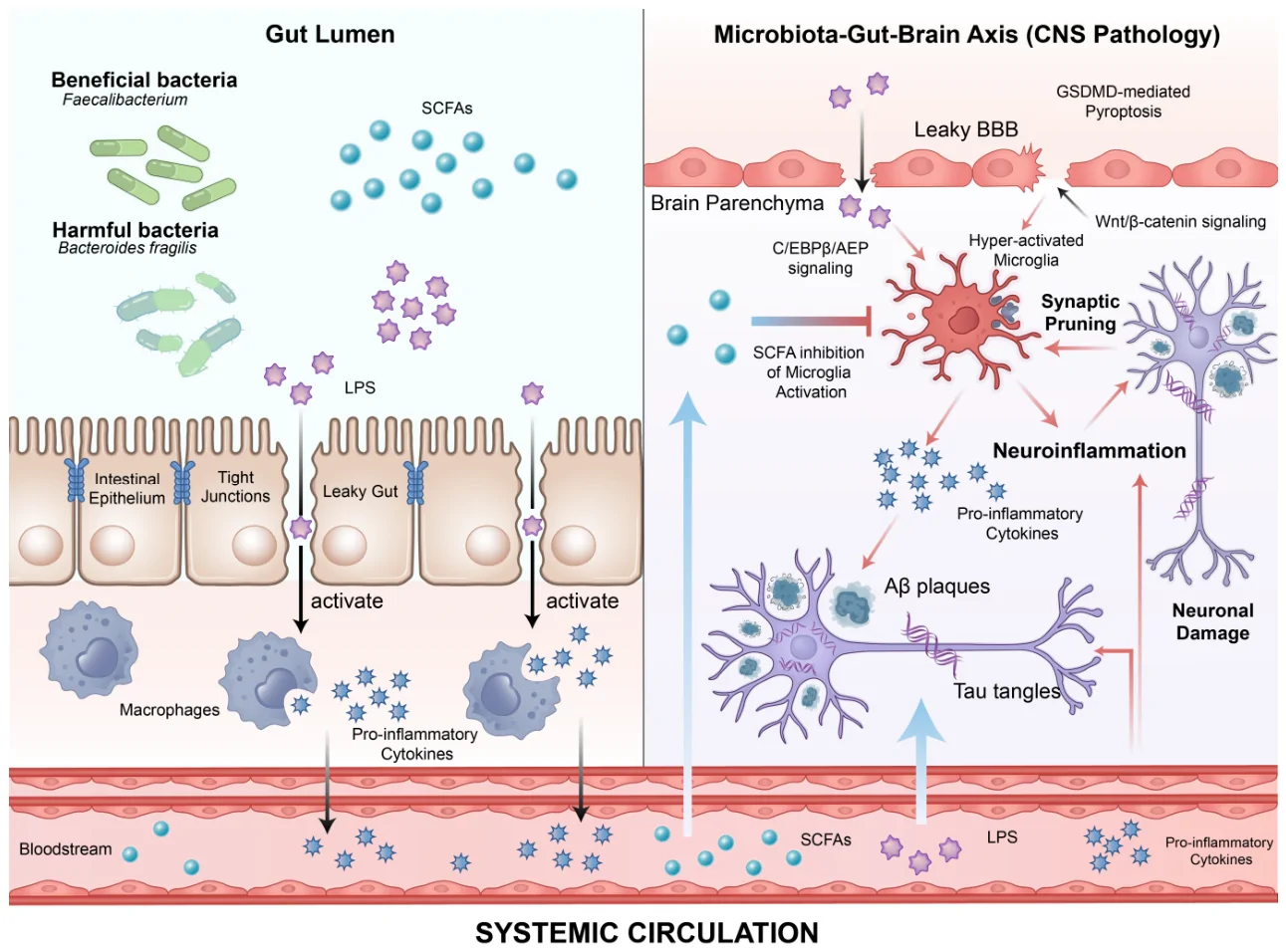
Multiple regulatory pathways of gut microbiota participate in the pathogenesis of Alzheimer’s disease (AD). Schematic diagram illustrating the regulatory effects of gut microbial metabolites and the inflammatory cascade mediated by gut dysbiosis in AD. Abbreviations: Aβ, amyloid-β; BBB, blood–brain barrier; C/EBPβ/AEP, CCAAT/Enhancer-Binding Protein β/Asparagine Endopeptidase; CNS, central nervous system; GSDMD, gasdermin D; LPS, lipopolysaccharide; SCFAs, short-chain fatty acids; Tau, microtubule-associated protein tau; Wnt/β, wingless-related integration site/β-catenin. Created by the author.

Of note, in a human observational study of community-dwelling elderly, SCFAs-producing bacteria such as *Bacteroides massiliensis* and *Bifidobacterium pseudocatenulatum* show independent associations with better cognitive performance. These bacteria exhibit highly active metabolic activities in the catabolism and utilization of carbohydrates and amino acids [55] (Figure 2).

Lipid-related metabolites also play important roles in the pathological progression of AD. A human meta-analysis has shown that as a microbial metabolite of choline and carnitine, elevated plasma levels of trimethylamine N-oxide (TMAO) are significantly associated with cognitive impairment. In animal models, its mechanisms may involve the activation of the NLR family pyrin domain containing 3 (NLRP3) inflammasome, promotion of neuroinflammation, and impairment of blood–brain barrier (BBB) integrity [56,57]. Conversely, some bacteria-derived lipids have neuroprotective effects. For instance, in animal and cell studies, lysophosphatidylcholine (LPC) produced by Bacteroides can activate the GPR119 receptor on neurons, effectively inhibiting neuronal ferroptosis by upregulating key antioxidant proteins such as glutathione peroxidase 4 (GPX4, EC 1.11.1.9), thereby alleviating pathological damage in AD [58]. In addition, disorders of lipid metabolism caused by gut dysbiosis, especially imbalanced polyunsaturated fatty acid (PUFA) metabolism, are a key driver of neuroinflammation [56,59]. In addition, other gut microbial products also participate in the regulation of AD. To be exact, secondary bile acids like ursodeoxycholic acid (UDCA) can exert anti-inflammatory and neuroprotective effects by activating the farnesoid X receptor (FXR) [59]. Similarly, tryptophan-derived microbial metabolites like indole derivatives can modulate the functions of microglia and astrocytes through activating the aryl hydrocarbon receptor (AhR), influencing neuroinflammation and synaptic plasticity [60].

### 5.2. Immune Pathway: Inflammatory Cascade Mediated by Gut Dysbiosis

As the largest immune organ in the human body, the intestine relies on gut microbiota homeostasis to maintain systemic immune balance. Gut dysbiosis can disrupt intestinal barrier integrity and trigger low-grade systemic inflammation. Subsequently, the signal conducts to the CNS and induces neuroinflammation, forming an inflammatory cascade from the intestine to the system and then to the CNS (Figure 2).

Under healthy conditions, gut microbiota and its metabolites like butyrate can effectively sustain the integrity of intestinal epithelial tight junctions. When dysbiosis occurs, intestinal barrier function is damaged, allowing microbial-associated molecular patterns such as bacterial LPS to translocate into the portal circulation. As a major component of the outer membrane of Gram-negative bacteria, LPS derived from Bacteroides fragilis exhibits particularly strong proinflammatory activity and can potently activate the NF-κB pathway in peripheral immune cells [9]. This consistent low-level endotoxemia will lead to a chronic, systemic inflammatory state. A human observational study provides direct clinical evidence. The distinct gut microbiota composition in patients with AD is significantly associated with altered levels of specific proinflammatory cytokines, endotoxins, and endothelial dysfunction markers in the blood. Also, these peripheral inflammatory mediators are further specifically linked to cerebral Aβ and Tau pathological burdens in patients, strongly supporting the regulatory pathway from gut dysbiosis to systemic inflammation and ultimately to central pathology [61].

Systemic inflammation can disrupt the BBB through multiple mechanisms. In animal and cell studies, LPS can directly induce caspase-4/11-mediated gasdermin D (GSDMD)-dependent pyroptosis in brain endothelial cells, causing structural BBB damage [62]. Meanwhile, LPS or proinflammatory cytokines can activate the endothelial NF-κB pathway, directly inhibiting the Wnt/β-catenin signaling pathway that is essential for BBB integrity. Consequently, it can downregulate the expression of tight junction proteins like ZO-1 and weaken BBB function at the transcriptional regulatory level [63].

In addition, peripheral inflammatory signals can be sensed and transmitted via innate immune receptors expressed on brain endothelial cells like Toll-like receptors, activating the microglial NLRP3-GSDMD axis. This axis upregulates the expression of chemokines and matrix metalloproteinases through mediators such as GDF-15, thereby actively exacerbating BBB injury [64,65,66,67,68]. Following BBB damage, consistent activation of microglia and astrocytes is further promoted, forming a self-amplifying loop of neuroinflammation [64]. Animal studies have shown that gut microbiota-driven Aβ pathology strictly depends on microglial participation [69]. Abnormally activated microglia via the C/EBPβ/AEP pathway not only exhibit reduced efficiency in Aβ clearance but also release large amounts of proinflammatory factors and reactive oxygen species, further damaging neurons [70,71]. One study found that *Bacteroidota* can inhibit the phagocytic clearance of Aβ by microglia through its metabolites or components and promote amyloid plaque deposition, which can accelerate AD pathological progression and provides direct evidence for the mechanism [72].

More importantly, in cell and animal studies, activated microglia can induce astrocytes to transform into neurotoxic reactive astrocytes, characterized by upregulation of complement component C3, which mediates excessive synaptic pruning and loss, directly leading to cognitive impairment [73]. Recent studies in animal models have also revealed that bacterial outer membrane vesicles released by gut bacteria can directly carry LPS into the brain and promote microglia-mediated synaptic pruning, uncovering a more direct pathway through which the microbiota affects the central nervous system [74]. A recent groundbreaking study identified a specialized “somatic-brain” neural circuit in the brain that actively monitors and suppresses excessive systemic inflammation, revealing a superior brain-dominated immunoregulatory mechanism within the gut–microbiota–brain axis [75].

### 5.3. Neuroendocrine Pathway: Synergistic Effects of Direct Neural Transmission and Stress Regulation

In addition to indirect metabolic and immune pathways between the gut and the brain, there are also two direct pathways, comprising direct transmission via the vagus nerve and systemic endocrine regulation through the hypothalamic–pituitary–adrenal (HPA) axis. Both pathways are jointly involved in microbiota-mediated regulation of AD.

#### 5.3.1. Direct Vagus Nerve Transmission Pathway

The intestinal wall contains an extensive enteric nervous system, known as the “second brain”. Gut microbiota and their metabolites like SCFAs can directly stimulate enterochromaffin cells to release serotonin (5-hydroxytryptamine, 5-HT), which acts on specific receptors on enteric nerve terminals, thereby activating afferent fibers of the vagus nerve [76]. These signals are rapidly transmitted to the nucleus tractus solitarius in the brainstem, which in turn regulates the function of higher brain regions related to emotion, cognition, and stress responses [76]. Animal studies have confirmed that resection of the vagus nerve can block the regulatory effects of certain microbiota interventions on brain function and behavior [77]. At the molecular level, vagus nerve activity can regulate the expression of brain-derived neurotrophic factor (BDNF) in the brain in animal models, which is critical for neural plasticity [78]. Interventional studies have illustrated that vagus nerve stimulation can improve intestinal barrier and BBB integrity by regulating the function of intestinal mast cells, providing indirect experimental evidence for AD’s precise intervention [79].

#### 5.3.2. The HPA Axis Endocrine Regulation

**Figure 3:**
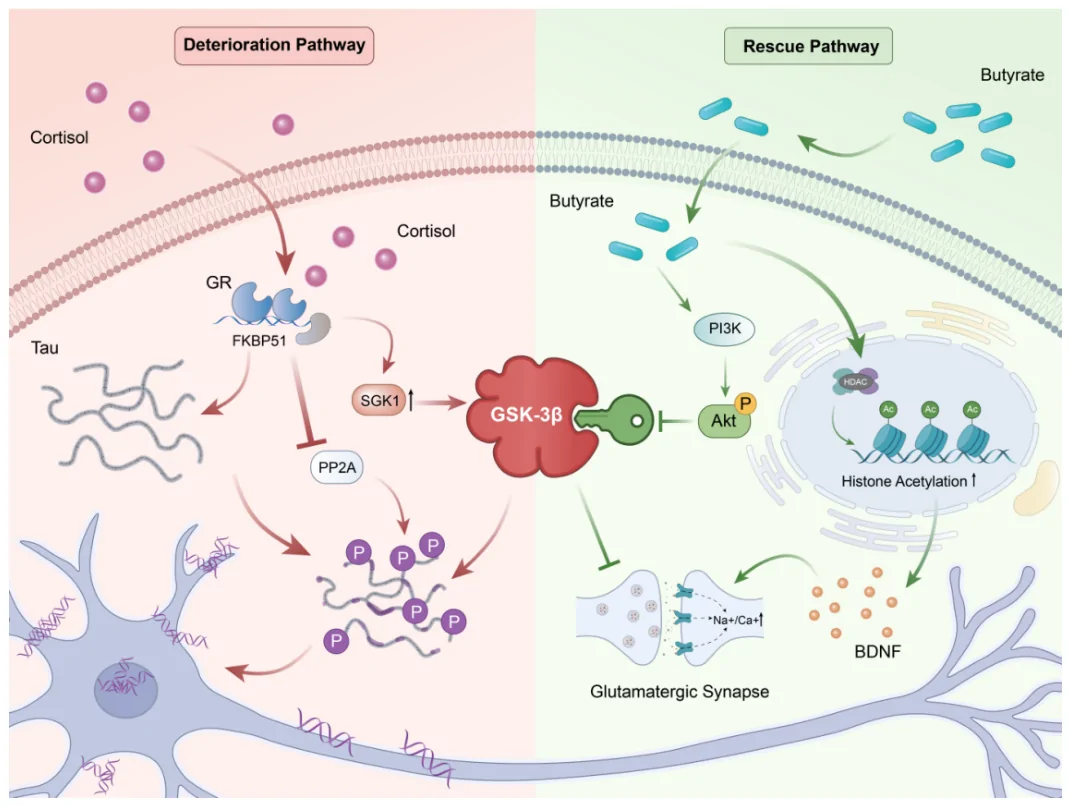
Bidirectional regulation of Alzheimer’s disease occurs via cortisol and butyrate. Schematic diagram demonstrating the deteriorative effect of cortisol and the neuroprotective effect of butyrate on neurons. Abbreviations: Akt, protein kinase B; BDNF, brain-derived neurotrophic factor; FKBP51, FK506-binding protein 51; GR, glucocorticoid receptor; GSK-3β, glycogen synthase kinase-3 β; PI3K, phosphatidylinositol 3-kinase; PP2A, protein phosphatase 2A; SGK1, serum and glucocorticoid-regulated kinase 1; Tau, microtubule-associated protein tau. Created by the author.

Gut microbiota is also involved in regulating the development and stress responses of the HPA axis [80,81]. Microbiota dysbiosis can impair the feedback regulation of the HPA axis in response to stress stimuli, leading to chronically elevated cortisol levels [81,82]. A prolonged high-cortisol environment exerts multiple neurotoxic effects on the hippocampus. A human clinical study have confirmed that chronic cortisol exposure, such as in Cushing’s disease, may give rise to reduced hippocampal volume, particularly in specific subregions, and is significantly associated with cognitive decline and abnormal connectivity in emotion-related brain networks [83]. Mechanisms include suppressed neurogenesis, impaired glutamatergic synaptic plasticity, and direct activation of glycogen synthase kinase 3β (GSK-3β, EC 2.7.11.26) [80]. Animal and human evidence indicates that GSK-3β is a key kinase driving excessive phosphorylation of Tau protein, while gut microbial metabolites such as butyrate can regulate GSK-3β activity through acetylation modifications, finally influencing Tau phosphorylation and its spread in the brain [84]. This process is a core molecular part of gut dysbiosis to the HPA axis hyperactivity, hypercortisolemia, and then to exacerbated Tau pathology (Figure 3).

### 5.4. Interacting and Synergistic Effects Among Mechanisms

The metabolic, immune, and neuroendocrine pathways are not independent or parallel processes, but rather intersect and interact synergistically at multiple levels, collectively influencing the pathological progression of AD. The interactions between these pathways can be reflected in the pathological process mediated by gut microbiota. Gut dysbiosis disrupts intestinal barrier integrity and induces systemic inflammation, which represents the immune pathway as supported by human observational evidence [9,61,64]. Such inflammatory insult further disturbs cerebral iron and lipid metabolism, impairs astrocytic lipid transport, and exacerbates neuronal ferroptosis, which constitutes the metabolic pathways shown in animal models and cell and animal studies [85,86,87]. Meanwhile, inflammatory stress triggers HPA axis hyperactivation, elevates cortisol levels, and enhances GSK-3β-mediated Tau hyperphosphorylation, reflecting the neuroendocrine pathway as reviewed and supported by animal and human evidence [80,84]. These three pathways mutually reinforce each other and create a self-amplifying cycle that accelerates AD progression. For instance, gut microbiota modulates cerebral iron homeostasis via ferritin and iron regulatory proteins [85]. In cell and animal studies, it also modulates lipid peroxidation and neuronal ferroptosis, while microbiota dysbiosis reshapes astrocytic cholesterol transport to alter neuronal membrane integrity and Aβ production [86,87].

The glymphatic system formed by astrocytic processes is surrounded by cerebral blood vessels and serves as the critical pathway for clearing metabolic waste such as Aβ in the brain under physiological conditions. This perivascular waste-clearing pathway differs from meningeal lymphatic drainage mediated by genuine lymphatic vessels. Studies indicate that chronic neuroinflammation and abnormal activation of microglia may damage the functional integrity of this system, leading to reduced Aβ clearance efficiency. Intestinal microbiota interventions like probiotics have been demonstrated to ease AD-related pathology by modulating systemic and intracerebral immune-inflammatory states in animal models. However, their direct beneficial effects on glymphatic function require further investigation [88].

## 6. AD Treatment Strategies Targeting Gut Microbiota

**Figure 4:**
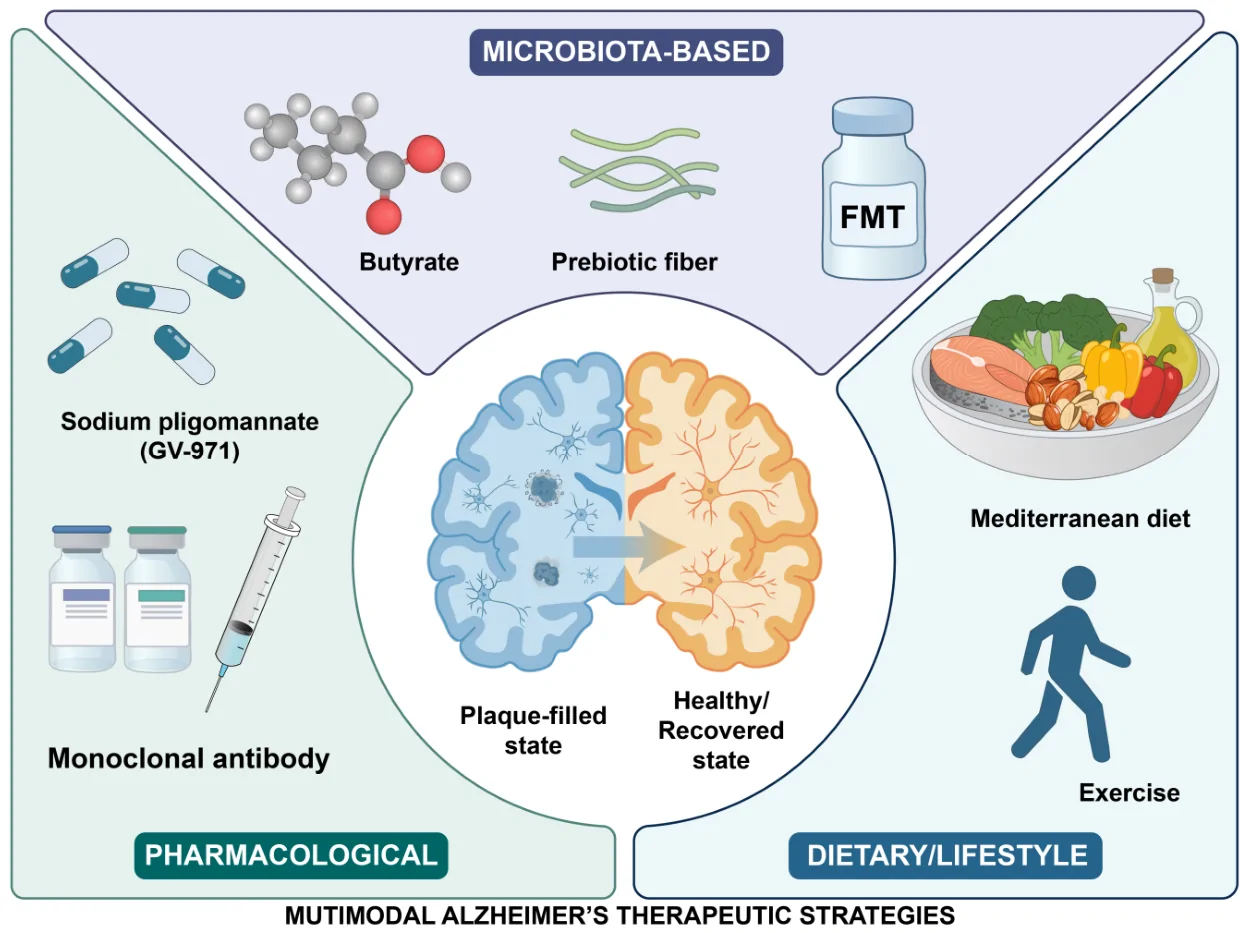
Multimodal therapeutic strategies are available for Alzheimer’s disease. Schematic diagram summarizing three main intervention approaches for Alzheimer’s disease. Abbreviations: FMT, fecal microbiota transplantation; GV-971, sodium oligomannate. Created by the author.

A variety of microbiota-targeted therapeutic strategies for Alzheimer’s disease can be classified into three categories. Microbiota-based approaches include butyrate, prebiotic fiber and FMT (fecal microbiota transplantation). Pharmacological treatments consist of GV-971 (sodium oligomannate) and monoclonal antibodies. Dietary and lifestyle strategies involve the Mediterranean diet and exercise (Figure 4).

### 6.1. Intervention Principle and Relevant Evidence

The gut microbiota has emerged as a prospective target for intervening in the pathological progression of AD. Current intervention strategies primarily rely on four fundamental mechanisms. Direct supplementation is the immediate introduction of beneficial functional units by probiotics like Bifidobacterium breve A1 or postbiotics like metabolites of microbial cells. Preclinical evidence, mainly from animal studies, supports their neuroprotective potential, although human evidence remains limited [51]. The second aspect is nutritional support. This approach selectively nourishes beneficial bacteria through dietary components such as prebiotics, thus promoting the production of protective metabolites like SCFAs. For example, inulin prebiotics can reshape the gut microbiota in AD model mice and increase levels of beneficial metabolites such as SCFAs and indole-3-propanoic acid, improving related disease phenotypes [89]. The next approach is ecological reset, represented by FMT, whose aim is the overall transplantation of a healthy microbial ecosystem. Animal studies have demonstrated that the microbiota from healthy donors can reduce Aβ and Tau pathology in AD mice, whereas AD-associated microbiota impair memory function in healthy mice, highlighting the essential role of microbial ecology in maintaining health [50,90,91,92,93]. The last approach is indirect regulation, which involves long-term shaping of beneficial microbiota through specific dietary patterns, exercise, and stress management [94]. For instance, both the modified Mediterranean diet and the modified Mediterranean ketogenic diet have been demonstrated to influence AD biomarkers or cognitive function by adjusting the microbiota and its associated metabolites [31,95] (Table 2).

### 6.2. Current Status of Clinical Translation: Heterogeneity and Positive Evidence

Although preclinical studies have accumulated substantial evidence, the translation to human clinical trials reveals significant individual heterogeneity in therapeutic efficacy. These differences are primarily influenced by host genetic background, disease stage, and gender [36,44,46]. However, multiple clinical studies have still provided positive signals. A multi-domain randomized controlled trial involving patients with early-stage AD demonstrated that comprehensive interventions can improve cognitive function and modulate gut microbiota [96]. Nevertheless, the effect size and clinical significance of these improvements require further validation.

More importantly, GV-971, a therapeutic agent targeting gut microbiota, was tested in a 36-week, multicenter, randomized, double-blind, placebo-controlled phase III trial in patients with mild-to-moderate AD [34]. The primary endpoint was the change in ADAS-cog12 from baseline to week 36, and the reported between-group difference was approximately two points. Adverse events were generally comparable to placebo [34]. However, cognitive improvement alone does not establish microbiota-mediated efficacy, because the trial did not measure gut microbiota or perform mediation analysis linking microbiota changes to cognitive outcomes. The phase-III trial was conducted only in China, and mechanistic observations from preclinical animal studies still await further corroboration in human cohorts [12,34]. Furthermore, its phase-III data and reproducibility have been debated internationally, requiring further independent multi-region validation.

### 6.3. Current Challenges and Future Directions for Precision Intervention

Current strategies for gut microbiota intervention in AD still face core challenges such as insufficient standardization and neglect of individual heterogeneity. A multidimensional framework on information integration should be created to promote precision medicine in the future. Experts should comprehensively consider patients’ ATN biomarker staging, specific gut microbiota ecotypes, and host genetic characteristics to achieve personalized intervention of AD. For example, for individuals in the preclinical stage only with Aβ pathology, dietary modification should be prioritized to shape and protect the ecosystem of beneficial microbiota [31]. For AD patients with significant neuroinflammation, more personalized intervention on specific targets may be required. Supplementation with selected next-generation probiotics capable of producing anti-inflammatory metabolites like butyrate may be seriously considered, though clinical evidence remains limited [97]. Cutting-edge technologies such as engineered bacteria have been explored for programmable metabolic and immune modulation in oncology [98]. Additionally, engineered butyrate-producing yeasts have shown benefit in AD animal models [54]. Phage-based approaches allow selective depletion of gut bacteria in mouse models, but their therapeutic efficacy in AD remains unestablished [99]. These approaches, together with personalized interventions based on ATN staging, microbiota ecotypes, and host genetics, should be framed as future research directions rather than established therapies for personalized AD intervention.

## 7. Conclusions

In this study, we reviewed the typical changes in gut microbiota in Alzheimer’s disease and analyzed how altered microbial communities relate to AD progression. This work further confirms the association between gut dysbiosis and AD pathogenesis and emphasizes the important role of the gut–brain axis in neurodegeneration. Our findings add supportive evidence for the microbiota-AD relationship and provide new clues for understanding AD development. However, current research still has several limitations and unresolved translational problems.

While gut microbiota-AD research has achieved remarkable progress, the field remains in a transitional phase, moving from observational association toward causal understanding and clinical translation [10]. Two core challenges currently limit advancement. First, most human studies only establish correlation, leaving causal inference weakly supported [32]. Second, a substantial translational gap persists, alongside limited reproducibility in clinical investigations. For example, FMT from AD patients into animals induces cognitive deficits, indirectly confirming the pathogenic potential of dysbiosis [50]. Yet how to reliably translate such animal findings into clinical practice remains a pressing unmet need.

The next wave of AD research must address these barriers from multiple angles. Efforts should focus on building large prospective stratified cohorts to validate causal mechanisms and on confirming the causal roles of specific bacteria in controlled models. Clinical translation should be streamlined by prioritizing agents with well-defined components and mechanisms. Sensitive biomarkers can serve as intermediate endpoints to accelerate trial timelines [32]. Building on these foundations, interdisciplinary collaboration will be essential for translating mechanistic insights into clinical practice. Specific precision intervention strategies are discussed in Section 6.3.

Beyond large prospective human cohorts, multi-omics integration, including metagenomics, metabolomics and transcriptomics, and multi-modal biomarker combinations will facilitate translational research linking gut microbiota to AD. Multi-omics approaches can capture multi-layer molecular alterations, while combined biomarkers may improve diagnostic performance for AD in clinical settings [100].

## Figures and Tables

**Table 1:** Summary of representative human observational studies investigating gut-microbiota alterations across the Alzheimer’s disease clinical spectrum.

Clinical Condition	Sample Size and References	Sequencing Method	Confounder Adjustment	Microbial Taxa	Change Direction	Key Findings	Limitations
Preclinical AD	[5]: *n* = 104	16S rRNA	Age, sex	Bacteroides	↑ ^1^	Microbiota dysbiosis appears before cognitive decline; related inflammatory changes	Cross-sectional; small sample; results need replication
Mild cognitive impairment (MCI)	[5]: *n* = 204; [35]: *n* = 147; [41]: *n* = 112	16S/shotgun metagenomics	Age, sex, BMI	*Faecalibacterium*, *Roseburia*	↓ ^2^	Reduced butyrate-producing taxa	Cross-sectional; medication not fully controlled; results not fully replicated
				*Escherichia*, *Shigella*	↑ ^1^	Increased proinflammatory bacteria	
Mild-to-moderate AD	[5]: *n* = 126; [7]: *n* = 40 (amyloid-positive); [38]: *n* = 89 (AD = 25)	16S rRNA	Age, sex	*Faecalibacterium*, *Roseburia*	↓ ^2^	Lower butyrate producers and microbial diversity	Cross-sectional; cannot rule out reverse causation; study heterogeneity
				*Escherichia*, *Shigella*	↑ ^1^	Enriched proinflammatory taxa associated with cognitive decline	
Advanced AD dementia	Multiple cohorts	16S/metagenomics	Variable	*Mixed taxa*	Inconsistent	Some cohorts report reduced diversity; others show no taxonomic changes	High heterogeneity; medication/comorbidities; conflicting published results

^1^ Upward arrows (↑) represent increased abundance of microbiota. ^2^ Downward arrows (↓) represent decreased abundance of microbiota.

**Table 2:** Summary of gut-microbiota-targeted clinical intervention studies for Alzheimer’s disease.

Intervention Type	Intervention Details	Microbial Effect	Main Outcomes
Sodium oligomannate (GV-971)	Oral oligosaccharide drug	Remodeled overall gut microbiota	Improved primary cognitive endpoint; secondary endpoints non-significant; China-only trial; preclinical mechanisms unvalidated in humans
Probiotic supplementation	Multi-strain probiotic capsules	Modulated gut microbial composition	Inconsistent cognitive benefits; limited evidence for AD
Fecal microbiota transplantation (FMT)	Fecal microbiota transfer from healthy donors	Restoration of recipient gut microbiota	Promising preclinical AD results; no robust large human RCT evidence
Dietary/multidomain lifestyle intervention	Mediterranean-style diet; multidomain intervention (cognitive training, exercise, nutrition)	Shifted microbiome (enriched *Bifidobacterium*, *Faecalibacterium*)	Multi-domain intervention normalized microbiome and improved cognition; pure dietary evidence weak for AD

## Data Availability

No new data were created or analyzed in this study. Data sharing is not applicable to this review.

## Abbreviations

AD	Alzheimer’s disease
Aβ	amyloid-β
BBB	blood–brain barrier
CNS	central nervous system
GSK-3β	glycogen synthase kinase 3β
HPA	hypothalamic–pituitary–adrenal
LPS	lipopolysaccharide
SCFAs	short-chain fatty acids
